# Estimating the Mobility of the Michaelis Sacral Rhombus in Pregnant Women

**DOI:** 10.7759/cureus.7116

**Published:** 2020-02-27

**Authors:** Marco Siccardi, Cristina Valle, Valentina Angius, Fiorenza Di Matteo

**Affiliations:** 1 Obstetrics and Gynaecology, Primal Osteopathy Institute, Savona, ITA; 2 Obstetrics and Gynaecology, San Paolo Hospital, Savona, ITA; 3 Yoga and Cranial Osteopathy, Primal Osteopathy Institute, Savona, ITA

**Keywords:** pelvimetry, dystocia, parturition, pelvis, anatomy, topographical anatomy, movement, sacrum, prevention, measures

## Abstract

Pelvic mobility is the cornerstone of an adequate birth canal for safe childbirth, and midwives invite pregnant women to assume loading positions to facilitate delivery. Biomechanics asserts that pelvic space changes in shifting positions from erect to the squat position. The current standard practice in obstetrics and osteopathy provides a qualitative observational assessment of the dimension of Michaelis sacral rhombus in shifting positions; a previous report presented a clinical method and instrument to estimate the pelvic range of motion through finger contact on bone landmarks. The present study aims to match the measurement of the diameters of the sacral area of Michaelis from skin marks with the amount from bone landmarks. Methods estimate the sacral area from 100 pregnant women in the late trimester, considering the dimension of the diameters, the range of motion, and the patterns of mobility. Differences resulted in the methods: measuring the skin marks in shifting positions revealed a not significant difference between starting position and squat position. The measurements through the finger contact on the bone landmarks seem to be adequate to estimate pelvic mobility fulfilling the expectation from biomechanics literature.

## Introduction

Since the beginning of obstetric pelvimetry, clinical studies have correlated the static dimension of the external diameters to the obstructed labor risk and the diagnosis of the contracted pelvis [1-4]. The “contracted pelvis” is a pelvis that has no or little articular and soft tissue mobility. In more recent years, interest appeared in the biomechanical study of the pelvis to elucidate the anatomic physiology of the birth process and better understand the mechanical dystocia from the contracted pelvis [5-11]. A clinical screening or diagnostic method addressed to the contracted pelvis, evaluating the range of pelvic mobility, would improve the quality of childbirth for mothers and babies. The sacral diamond-shaped area of Michaelis is part of the assessment of obstetric pelvimetry, related to the pelvic inlet arrangement and disposition, where the fetal head negotiates the passage. It consists of a rhomboid shape area set by four sides, a transverse and a longitudinal diameter [12].

The change in dimensions of the sacral area observed in shifting positions is widely considered in the obstetric and osteopathic ﬁeld the current standard indicator of pelvic health in pregnancy for the performance of safe and natural childbirth [13-18]. Midwives noted that during the second stage of labor, the sacral area progressively expands its transverse and longitudinal diameters. They suggest assessing the Michaelis rhombus, signed by skin marks, during the pregnant period through the observation (or palpation) of the sacral area in shifting maternal positions from erect to the squat position [13,14]. The available bibliography about the observational evaluation of the sacral area refers to a subjective assessment of the quality of motion, failing to deﬁne the amount of the enlargement observed, not measuring the distance between the marks drawn on the skin [13-18]. Moreover, the skin is not firmly attached to the bones, so the observation of the skin marks could not correspond to the pelvic articular mobility. Some studies showed that the skin markers give systematic errors due to the soft tissue artifacts occurring during movements [19,20]. We have proposed a novel method of dynamically measure the diameters of the external pelvimetry through the operator's hands used as contact trackers on the bone landmarks [11]. The biomechanics studies of the obstetric pelvic spaces in shifting positions, estimated by MR and optoelectronic tracker devices, are the foundation for the Dynamic External Pelvimetry (DEP) test. We hypothesized that the DEP test could meet pelvic mobility better than skin observation.

Our study aims to consider the measurement procedures, firstly estimating and quantifying the postural dynamic changes in dimensions of the transverse and longitudinal diameters of the sacral rhombus of Michaelis, measured by skin marks distance in three different standardized postures [17]. As a current clinical obstetric gold standard, we would match the skin marks measurements to the measurements obtained by finger contact on the bone landmarks, to check the effectiveness of the DEP test procedure and instrument [12].

## Materials and methods

This cross-sectional descriptive study was conducted in conformity to the Helsinki declaration on a cohort of 100 nulliparous women at the beginning of their third trimester of gestation (raw data deposit ﬁle doi:10.13140/RG.2.2.31565.79848). The first author, who refers to the Obstetrics Department of “San Paolo” Hospital of Savona, Italy, EU (institutional board approval number 1118, prot2019.119933), defined the study protocol. Data from patients admitted at the low-risk pregnancy clinic of the department between 30 January 2018 and 15 March 2019 were analyzed. Explained the study protocol and its aims, patients expressed their verbal informed consent to utilize data. The study accepted pregnant women who did not report abdominal, pelvic, or vertebral surgical interventions and did not have complaints at the clinical evaluation.

**Figure 1:**
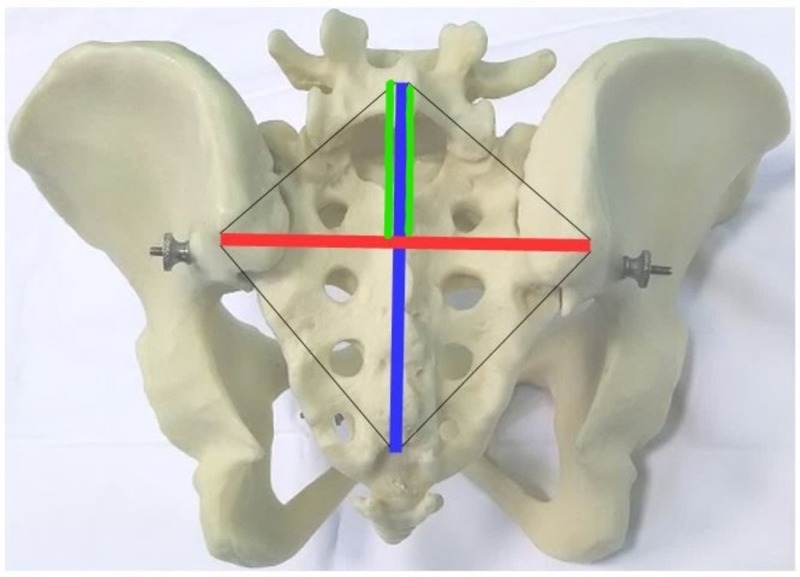
The Michaelis sacral rhombus. Redline: transverse diameter. Blueline: longitudinal diameter. Greenlines: hemi-longitudinal diameter.

As standard procedure, a dermographic pen drew the Michaelis sacral rhombus, deﬁned by the two posterior superior iliac spines (PSIS), by the spinous process of the ﬁfth lumbar vertebra (L5) and by the upper limit of the intergluteal fold corresponding to the fourth/ﬁfth sacral segment (S5) (Figure 1). The bump on the dimples overlying the gluteal region, at the posterior end of the iliac crest laterally to the sacrum, is the PSIS. The spinous process of L5 was identiﬁed in the following two ways. Firstly, by tracing a transverse line between the PSIS, the second sacral segment is identiﬁed, the ﬁrst sacral portion is at the top, space, and, therefore, the spinous process of L5. Then, by tracing a transverse line between the upper edge of the iliac crests, the space between the spinous processes of the 3rd and fourth lumbar vertebrae (L3 and L4) is identiﬁed, then the spinous process of L4 below, the space between L4 and L5 and therefore the spinous process of L5. By combining the two methods, the upper bone landmark of the sacral area is precisely deﬁned. The transverse diameter is the distance between the PSIS; the longitudinal diameter is the distance between the spinous process of L5 and the upper limit of the natal cleft.

**Figure 2:**
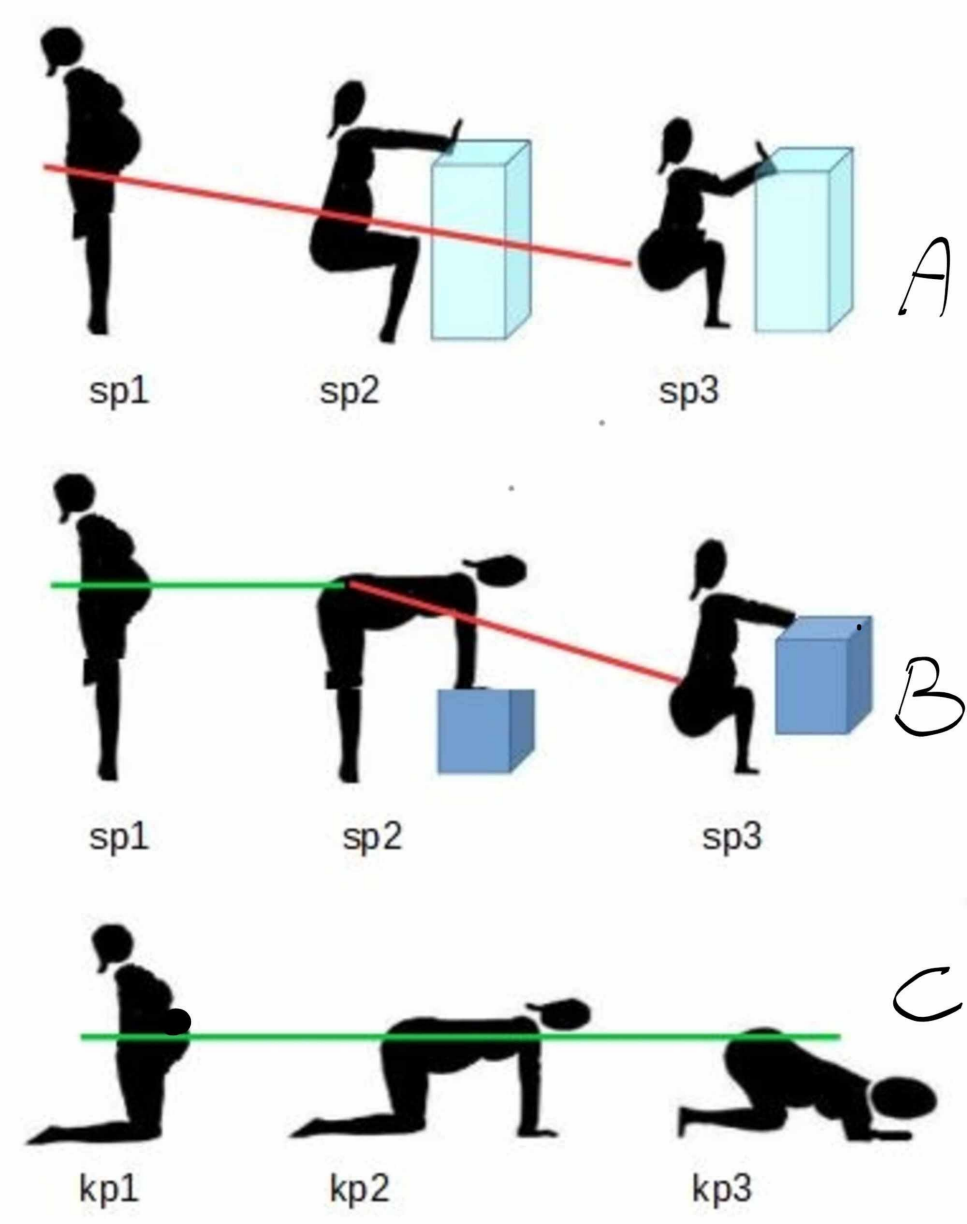
Postural methods of shifting positions. (A-B) Standing positions. (C) Kneeling positions. The redline and greenline highlight the level of the sacral area from the support during shifting positions. sp: standing position; kp: kneeling position.

We evaluated the transverse and longitudinal diameters of the Michaelis area in the following three positions deﬁned by previous studies, with the following postural change method (Figure 2), which revealed to be the more comfortable and safe for patients and operators [12,17].

- Position 1 (P1), “kneeling upright” situation: the patient is standing upright with support to the ground with her knees.

- Position 2 (P2), “hands-and-knees” supports: the patient ﬂexes the torso and also from the p1, carrying the weight on the arms with the support of the hands to the table.

- Position 3 (P3), “squat on the knee” (kneeling squat) position: the patient from the P2 still ﬂexes the hips bringing the ischial tuberosities towards the heels and the forehead towards the table.

**Figure 3:**
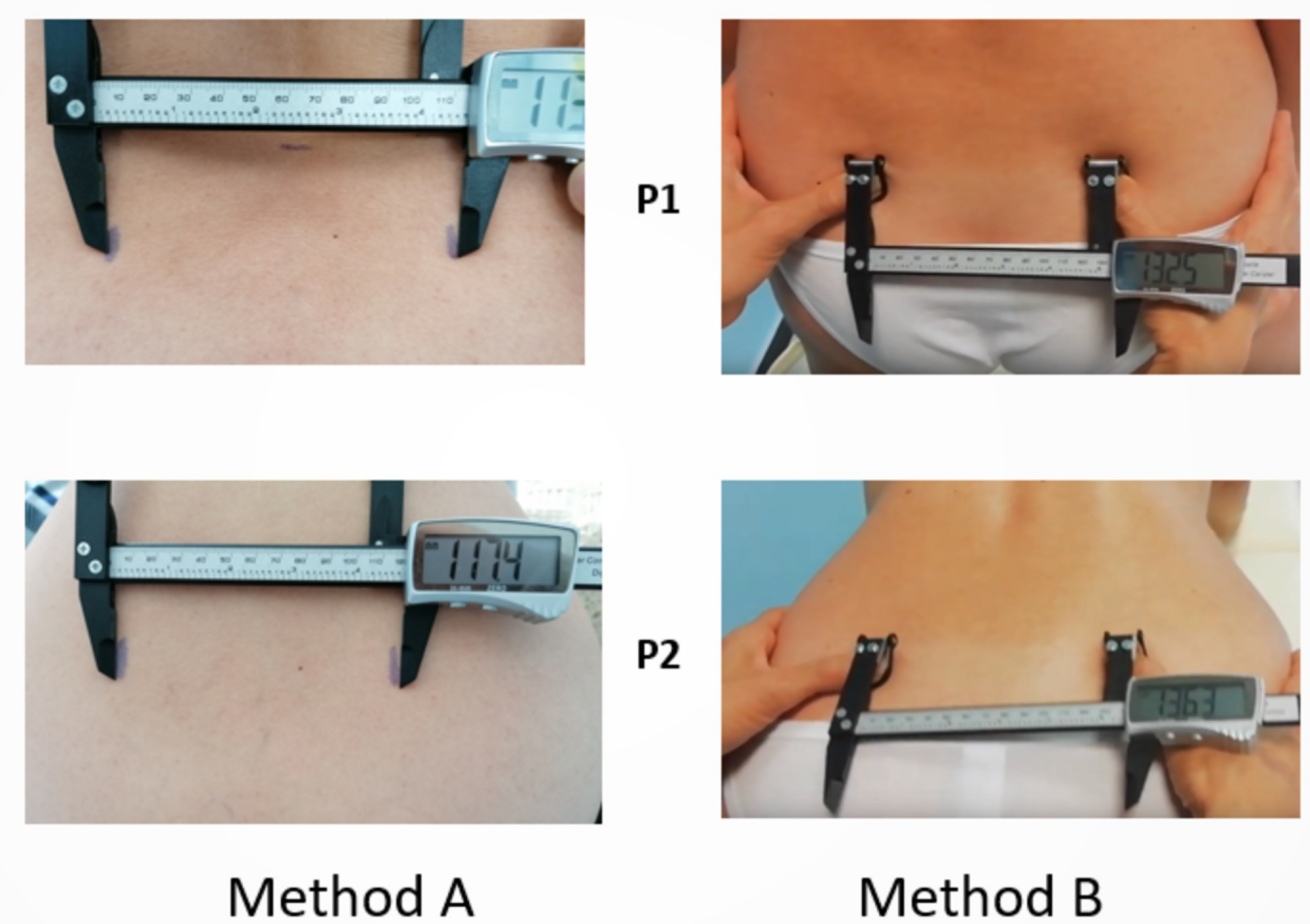
Measurement methods and instrumentation. The Bone-Meter Kit (BMK) instrument shows value in millimeters and tenth of a millimeter. Images from two different subjects in erect position (P1) and “hands-and-knees” position (P2).

Two methods were used for the quantitative measurement of the sacral diameters (Figure 3).

Method A (observational/skin marks measurements) measures the distance of the skin signs: for the transverse diameter, the SIPS-SIPS distance, for the longitudinal one, the L5-S5 range. The skin activates the elastic ﬁbres in shifting the positions, so the pen marks widen: we placed the lower beaks of the caliber on the outer edge of the skin marks for the measurement detection.

Method B (bone landmarks measurements by finger contact) measures the sacral diameters with the direct palpation of the ﬁngers on the bone landmarks, marked by the skin signs: the operator maintains steady contact while the subject is shifting positions [12].

All subjects performed the method of postural change five times: the ﬁrst time as evidence, and then alternating the measurement of the diameter of skin signs with the measure on bone landmarks.

All measurements were performed with the tool “Bone-Meter Kit” (BMK) (Metrica SpA, Milan, Italy, EU) ad-hoc designed and pending CE patented. The BMK is a precision caliber modiﬁed: the operator’s ﬁngers can wear it, via a top extension equipped with rings. The instrument has an accuracy of 0.2 mm and a resolution of 0.1 mm. The measurement of the distances between the two bony landmarks of each diameter occurs through their direct palpation by the phalanges of the operator’s ﬁngers wearing the BMK. The operator keeps the contact ﬁrmly and delicately on the anatomical points during the changing positions of the subject. A single measure for each method was stored and considered to emphasize errors in the agreement and precision of the measurements [21].

Open-source calculator Statstodo (Statstodo Trading PTY, Ltd, Brisbane, Queensland, Australia) performed the following statistical analysis: the normality Kolmogorov-Smirnov test, the Intraclass Correlation Coeﬃcients (ICC), the ANOVA test, the ANOVA test for repeated measures, and the two-paired sample Student's t-test for repeated measures. The reference table for the assessment of the ICC was: 0.0-0.2 indicates poor agreement; 0.3-0.4 indicates modest agreement; 0.5-0.6 indicates good agreement; 0.7-0.8 indicates strong concordance; >0.8 indicates an almost perfect agreement. An ICC value higher than 0.8, and a p-value <0.05 was considered statistically signiﬁcant.

## Results

The cohort of pregnant women showed (mean ± standard deviation) age 31.4 ± 5.6 years, height 162.8 ± 7.3 cm, weight 69.2 ± 11.1 kg; the evaluation was at 34.6 ± 2.7 weeks’ gestation. The mean and standard deviation of the transverse diameter is shown in Table 1. ICC calculations of the transverse diameter data showed quite a “perfect agreement” between the two measurement methods about the absolute value of dimensions in the erect and “all-four” positions, not in the squat position. The ANOVA test for the transverse diameter evaluation showed significant statistical differences between methods for measurements obtained in the “all-four” and squat position. The diameter evaluated by skin marks has narrower dimensions and a lower range of movement (Table 1).

**Table 1:** The dimensions in shifting positions of the transverse diameter of the Michaelis sacral rhomboid area obtained from the two methods. Data analysis performed between methods and between positions (P1, P2, P3: see the main text). Data expressed in millimeters as mean ± standard deviation. ICC: Intraclass correlation coeﬃcient; N.S.: Statistically not signiﬁcant.

Position	Method A	Method B	ANOVA p-value	ICC
P1	125.6 ± 15.06	126.02 ± 14.4	N.S.	0.96
P2	131.8 ± 14.7	134.1 ± 14.1	<0.0001	0.93
P3	125.4 ± 16.2	131.6 ± 15.8	<0.0001	0.77
Paired ANOVA p-value	<0.0001	<0.0001		

Moreover, the range of movement of the transverse diameter measured by skin marks exhibited a precise overlap of the measurements taken at the erect and squat positions (Table 2). Method B showed a statistically signiﬁcant difference in ROM between postures, revealed a larger diameter in the squat position than in the starting position. The ICC between the methods regarding the ROM of the transverse diameter indicated “poor agreement” (Table 2).

**Table 2:** Transverse diameter. The paired t-test of the difference between measurements in the different positions for Method A and Method B. ICC of the ROM between methods. Data expressed in millimeters as mean ± standard deviation (SD). 95% CI: 95% conﬁdence interval. ICC: Intraclass correlation coeﬃcient; ROM: Range of motion; Diff.: the difference between positions (P1, P2, P3: see the main text).

	Method A	Method B	Method A	Method B	Method A	Method B
	Diff. P2-P1	Diff. P2-P1	Diff. P3-P1	Diff. P3-P1	Diff. P3-P2	Diff. P3-P2
Mean (SD)	6.14 (4.3)	8.17 (4.4)	-0.23 (5.7)	5.63 (8.5)	-6.3 (7.2)	-2.54 (6.2)
95% CI	5.2–7.01	7.2–9.06	-1.3–0.9	3.9–7.3	7.8–4.9	3.7–1.2
p-value	<0.0001	<0.0001	N.S.	<0.0001	<0.0001	0.0001
ICC	0.36		0.30		0.26	

The absolute measurements of the longitudinal diameter had a “perfect agreement” for both methods, and the analysis of variance showed no difference (Table 3). The t-test of ROM in shifting positions was significant for both techniques (Table 4); the ICC of ROM indicated “good agreement” between methods ranging between 0.5 and 0.7, higher than the transverse diameter but lower than the signiﬁcance threshold.

**Table 3:** The dimensions in shifting positions of the longitudinal diameter of the Michaelis sacral area obtained from the two methods. Data analysis performed between methods and between positions (P1, P2, P3: see the main text). Data expressed in millimeters as mean ± standard deviation. ICC: Intraclass correlation coeﬃcient; N.S.: Statistically not significant.

Position	Method A	Method B	ANOVA p-value	ICC
P1	119.3 ± 16.5	120.5 ± 16.1	N.S.	0.89
P2	147.1 ± 14.8	148.7 ± 15.2	N.S.	0.85
P3	153.1 ± 18.6	153.1 ± 19.2	N.S.	0.89
Paired ANOVA p-value	<0.0001	<0.0001		

**Table 4:** Longitudinal diameter. The paired t-test of the difference between measurements in the different positions for Method A and Method B. ICC of the ROM between methods. Data expressed in millimeters as means (standard deviation). 95% CI: 95% confidence interval. ICC: Intraclass correlation coefficient; ROM: Range of motion; Diff.: the difference between positions (P1, P2, P3: see the main text)

	Method A	Method B	Method A	Method B	Method A	Method B
	Diff. P2-P1	Diff. P2-P1	Diff. P3-P1	Diff. P3-P1	Diff. P3-P2	Diff. P3-P2
Mean (SD)	27.8 (9.1)	28.1 (11.1)	33.7 (11.9)	32.5 (13.9)	5.9 (9.8)	4.3 (12.1)
95% CI	25.6–29.9	25.5–30.8	30.9–36.5	29.3–35.8	3.6–8.2	1.5–7.2
t-test p-value	<0.0001	<0.0001	<0.0001	<0.0001	<0.0001	0.003
ICC	0.50		0.63		0.70	

The longitudinal diameter lengthened progressively from the erect to the squat position, not the transverse diameter. Four different ROM patterns of the transverse diameter in the shifting stance were detected, and the two methods show different rates (Tables 5, 6). In the ﬁrst model, Pattern One, the transverse diameter increases progressively between P1, P2, and P3 (p1 < p2 < p3 > p1). In the second scheme (Pattern Two), the diameter measured in squats is smaller than P2 but broader than the starting position (p1 < p2 > p3 > p1). Pattern Three: the width in P3 is less than P2 and P1 (p1 < p2 > p3 < p1). Pattern Four, the transverse diameter gradually decreases between P1, P2, and P3 (p1 > p2 > p3 < p1): this is the only pattern in which P2 diameter is smaller than P1.

**Table 5:** The four dynamic patterns of the transverse diameter measurements of the sacral area obtained with Method A (skin marks measurement). Data expressed in millimeters as mean ± standard deviation.

Pattern	Rate	P1	P2	P3
One	5%	133.2 ± 15.6	136.4 ± 14.5	140.8 ± 8.8
Two	46%	125.2 ± 14.01	133.2 ± 14.2	128.6 ± 13.8
Three	48%	124.7 ± 15.7	129.6 ± 15.2	120.3 ± 17.3
Four	1%	150	149	142

**Table 6:** The four dynamic patterns of the transverse diameter measurements of the sacral area obtained with Method B (finger contact on bony landmarks). Data expressed in millimeters as mean ± standard deviation.

Pattern	Rate	P1	P2	P3
One	36%	126.7 ± 13.6	136.6 ± 13.2	140.6 ± 13.1
Two	38%	123.7 ± 12.6	132.9 ± 12.4	128.6 ± 12.1
Three	24%	128.6 ± 18.5	133.6 ± 17.5	124.4 ± 18.9
Four	2%	124.5 ± 5.6	120.5 ± 4.9	113.5 ± 4.9

## Discussion

The current study is about the procedures estimating the dynamic change of the dimensions of the Michaelis sacral area, and it reports data from the skin markers measurement, which is the objectivation of the subjective observation of the pelvis, compared to measures from finger contact on the bone landmarks. The means of the diameters in erect position returned values corresponding to literature data of obstetric pelvimetry. The dimensions of the widths of the kite-shaped sacral area modified significantly in shifting positions, expressing four changing patterns. The data analysis showed quite an adequate correlation between the raw measures, but the methods were not superimposable for the ROM estimation. The range of motion and the rate of the four patterns were significantly different between modes (Tables 2, 4-6). Method A showed identical measurements between the extended hips and the squat position (Table 1), contradicting its assumption that the sacral area enlarges progressively from the erect posture and the literature data that affirm the more pelvic space in the squat position [5-8,11,13-18]. Studies explained the external landmarks move with body movement due to the soft tissue artifact, that would affect the stability and precision of the skin markers located above the bone landmarks during the shifting positions [19-20]. It was more evident for the transverse than longitudinal diameter motion: the evidence for the Schober's test revealing a good agreement between skin and bone in the flexing mobility of the sacrolumbar area, explained our findings [22]. Schober’s tests estimate the degree of lumbar and lumbar-sacral flexion by measuring the distance between two skin marks by a flexible meter.

The assessment by bone contact seems to correspond better to the evidence reported in the literature [5-8,11,13-18]: the ﬁngers of the operator wearing the instrument remain in constant and stable connection with the bone reference points as the patient moves from one position to the next, avoiding the superficial soft tissue artifacts. We had considered several studies that indicate errors in the reference position, focusing on the poor reliability of clinical trials involving the palpation of pelvic bone landmarks [23-25]. Recognizing the anatomical points of the pelvic bone and ﬁnding them constantly over time are among the possible biases of the method; therefore, we referred to the range of motion that would be independent of the exact point of measurement [23,12]. Regarding the aims of the study on obstetric external pelvimetry, ROM estimation could resolve the scarce accuracy of the manual palpation of the reference points, and the finger bone contact avoids the artifacts due to the superficial soft tissues.

The dynamic activities and alternative positions are encouraged by midwives during labor, and moving may generate greater pelvic mobility than the comparable static posture [26]. Some evidence suggested that the intermediate position (hands-and-knees position) has a better impact on the dimensions of the diameters of the birth canal than the supine or squat position and could resolve fetal malpositioning in labor [7-9]. Similarly, both methods recorded that the posture with the hip joints ﬂexion at 90 degrees favors the greater transverse diameter of the sacral area (Table 1). Method B showed a significantly higher dimension than Method A. The longitudinal diameter showed lengthening with both methods, as the degree of ﬂexion increases (Tables 3, 4).

Coming back to the basic midwifery assertions, a traditional saying of Jamaican midwives states that a woman has to open her back before giving birth [15,16]. The observed “opening of the back” could be postulated as an enlargement of the Michaelis lumbosacral area that occurs during childbirth. Biomechanics stated that shifting in the squat position, the sacral base moves posteriorly in contra-nutation, and the lumbar spine flattens moving into flexion. The ilia rotate anteriorly, and the space in between enlarges at the level of the posterior superior iliac spines (PSIS): they agree with an increase in the dimensions of the Michaelis area diameters [7,8,27].

The “rhombus” comprehends the lumbosacral junction, the sacroiliac joints, the “thoracolumbar composite” section of the thoracolumbar fascia [27-29]. Changing the dimensions of the sacral area in shifting positions engages the mobility of such anatomical structures, influencing the pelvic rooms of the birth canal. From the findings of our study, the skin marks observation seems to be less suitable for reflecting the posterior pelvic mobility than the reference to the bone landmarks.

**Figure 4:**
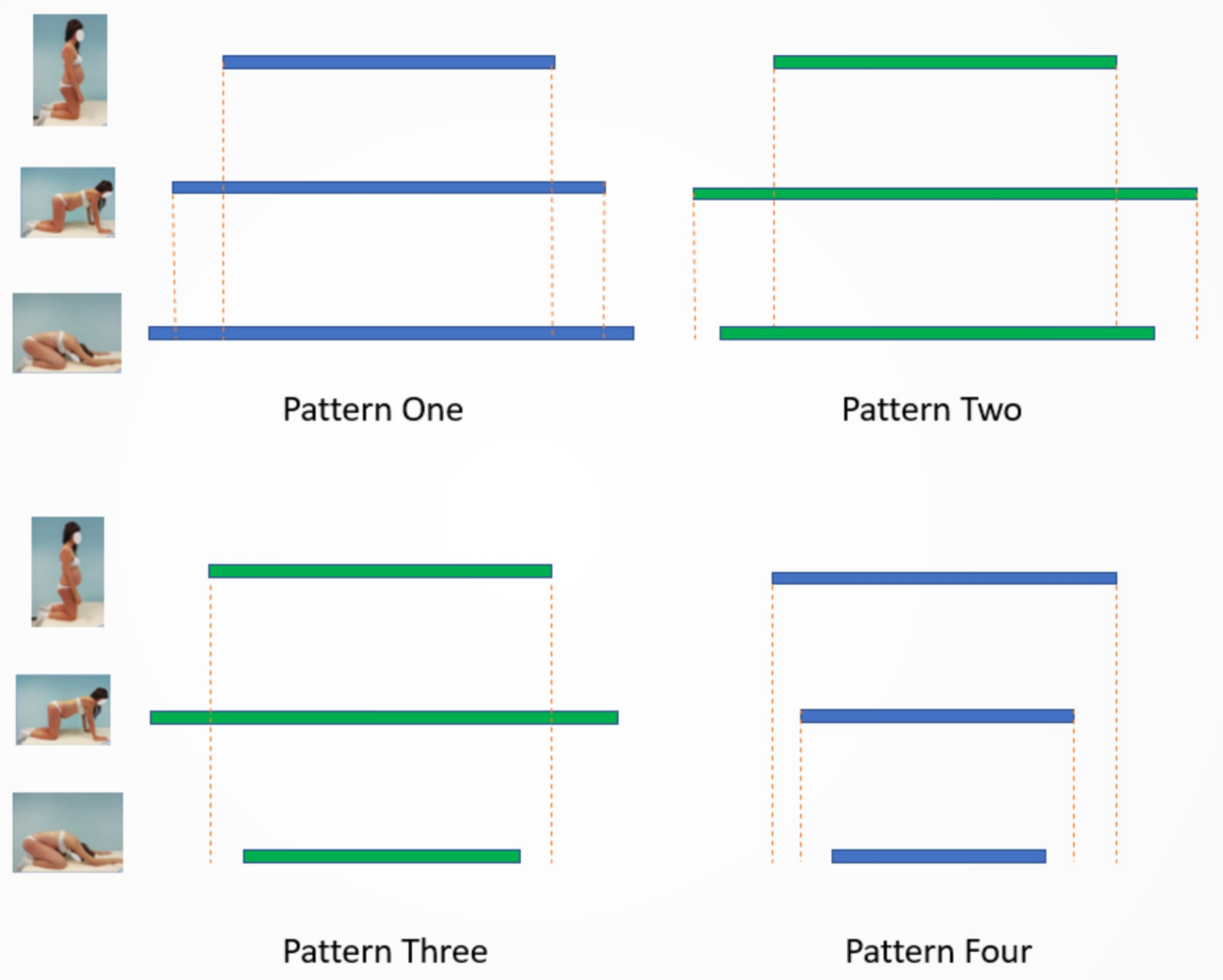
Graphical representation of the motion patterns of the transverse diameter in shifting positions. Bars represent the relative dimension of the transverse diameter in the position represented.

The present study gathered four different patterns of mobility of the transverse diameter (Figure 4), as the pelvic shape classes. We are not able to refer the movement patterns to the pelvic types of Caldwell and Moloy, which reported on a Caucasian population of 147 subjects the following proportions: gynoid, 41.4%; android, 32.5%; anthropoid, 23.5% and platypelloid, 2.6% [30]. However, identifying the scheme in which the pelvis moves can lead to detecting patients that enlarge all the spaces of the pelvis in the squat position from women that narrow the upper pelvis. It could improve the quality of assistance to the pregnant women in labor, supporting midwives to suggest the correct position during childbirth. Further studies need to elucidate the shapes of the diamond area, and patterns of mobility related to pelvic shape classes, and obstetric outcomes.

Skin marks revealed a progressive widening of the interiliac space in only 5% of patients. In 50% of subjects, the transverse diameter in the squat position is more extensive than in the erect position. Data seem to mean that visual subjective estimation is not able to evaluate the pelvis movement properly. Measurement by finger contact on the bone landmarks showed a 74% subjects transverse diameter enlarge in the squat than erect position. The DEP test for the Michaelis area, demonstrating broader ROM and higher values, seems more suitable than the usual observing procedure to be further studied as a clinical screening test for mechanical dystocia. It can be used for the mobility estimation of all obstetric external pelvimetry diameters in shifting positions [12].

## Conclusions

The present study on estimating the measuring procedures did not conﬁrm adequate changes in the shape of the sacral area evaluated by skin marks in shifting positions. The mere observation of the skin marks did not show a real increase in the transverse diameter of the sacral region in the squat position as expected. Fingers’ contact on bone landmarks met expectations from literature, is a safe and secure method, and could be useful for the dynamic postural assessment of the sacral area and the external obstetric pelvic diameters. Clinical studies will need to elucidate more in-depth the shape and motion of the Michaelis area, relate the ROM and its patterns to obstetric outcomes, and verify its possible use in the screening of obstructed labor from mechanical dystocia.
